# Arthroscopic Lysis of Adhesions With Adjuvant Radiation for Recalcitrant Knee Arthrofibrosis After Ligament Reconstruction: A Report of Three Cases

**DOI:** 10.1155/cro/4755736

**Published:** 2025-10-27

**Authors:** Louis Kang, Gavin Ward, Jason Ina, Paul Brown, Aaron J. Krych, Adam J. Tagliero

**Affiliations:** ^1^Department of Orthopedic Surgery, Mayo Clinic, Rochester, Minnesota, USA; ^2^Department of Radiation Oncology, Mayo Clinic, Rochester, Minnesota, USA

## Abstract

We report three patients—a 35-year-old female, a 19-year-old female, and a 36-year-old male—who presented with recalcitrant knee arthrofibrosis following anterior cruciate ligament reconstruction (ACLR) and prior lysis of adhesions (LOA). Each patient underwent a multistep treatment approach including revision arthroscopic LOA followed by low-dose irradiation, performed at 26, 8, and 6 months, respectively, after the index procedure. All patients experienced improved range of motion and better outcomes compared to prior interventions. In patients with recalcitrant arthrofibrosis after ACLR who have not responded to standard management, a revision LOA and low-dose irradiation can be safely performed, offering a moderate success rate for the most challenging cases.

## 1. Introduction

Arthrofibrosis is a complication of anterior cruciate ligament reconstruction (ACLR), affecting between 2% and 35% of patients [1, 2]. Its etiology is multifactorial, but it is understood to be triggered by an inflammatory response to trauma or chronic repetitive injury [3]. It can cause significant joint pain, stiffness, swelling, and limitation in range of motion (ROM), affecting activities of daily living (ADL). There is a significantly higher risk in women and patients with a history of prior surgery [3, 4]. Initial nonsurgical management of arthrofibrosis may include physical therapy (PT), continuous passive motion (CPM), or oral corticosteroids [3, 5]. If patients fail nonoperative measures, manipulation under anesthesia (MUA) is often considered in isolation or combined with an arthroscopic lysis of adhesions (LOA). Arthroscopic LOA is the primary surgical procedure for arthrofibrosis and has demonstrated efficacy in patients with recalcitrant ROM deficits following knee ligament reconstruction [6–8]. Previous studies reporting favorable outcomes describe arthroscopic LOA as typically being performed 2–20 months postoperatively after ligament reconstruction surgery [8–11].

Irradiation for the management of recalcitrant arthrofibrosis has rarely been reported, and the few existing studies focus on its implementation for total knee arthroplasty (TKA) [12–14]. Reported improvements in total arc ROM compared with preoperative values were 36° at 1-year follow-up, 52° at a mean of 2.8 years, and 60° at a mean 6-year follow-up in TKA patients [12–14]. Low-dose radiotherapy has shown efficacy in targeting overexpressed growth factors such as TGF-*β* and PDGF, excessively proliferating fibroblasts, and also modulating excessive inflammatory processes [15]. The objective of this study was to report the outcomes of three patients with recalcitrant arthrofibrosis following ACLR who underwent combined revision arthroscopic LOA and low-dose irradiation. The patients were informed that data regarding their cases would be submitted for publication, and consent was obtained.

## 2. Case Presentation

### 2.1. Case 1

A 35-year-old female developed right knee arthrofibrosis following an ACLR, lateral extra-articular tenodesis, and medial patellofemoral ligament reconstruction (MPFLR) at an outside institution 2 years prior. No meniscus repair was performed at the time of surgery. The patient subsequently underwent three LOA and MUA procedures with limited improvement, most recently 6 months prior. She underwent extensive PT, used dynamic flexion and extension braces, and was prescribed losartan. Upon presentation to our institution, the patient's ROM was 15° of extension to 75° of flexion (Table 1). Magnetic resonance imaging (MRI) revealed intact ACLR and MPFLR grafts without significant effusion or cyclops lesion.

**Figure 1:**
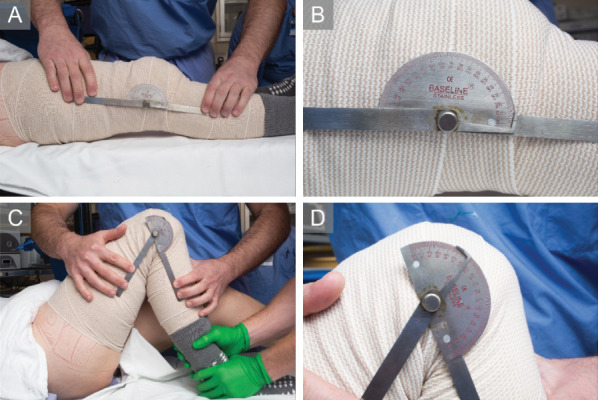
Range-of-motion measurements for Case 1, 35-year-old female, following lysis of adhesions and posterior capsular release, before irradiation therapy. (A) Extension to 5°, with a (B) zoomed-in view. (C) Flexion to 125°, with a (D) zoomed-in view.

The patient was offered a multimodal intervention including revision arthroscopic LOA, posterior capsular release (to address extension deficit), MUA, and one-time low-dose irradiation. Examination under anesthesia (EUA) revealed ROM of 15°–75°. Arthroscopy showed scar tissue in the suprapatellar pouch, proximal lateral trochlea, proximal medial gutter near the MPFLR, and deep to the infrapatellar fat pad. Following scar tissue debridement, a posterior capsular release was performed. MUA demonstrated improved ROM of 5°–125° (Figure 1). On postoperative Day 1, the patient received 800 centigray (cgy) of irradiation to the operative knee. The radiotherapy fields for this patient, typical for this indication, were opposed fields anteroposterior–posteroanterior (AP-PA) with the patient supine. In close collaboration with radiation oncology, the target was delineated, including the joint line and surrounding tissue. Additionally, the patient utilized a CPM device, turnbuckle orthoses, PT, and Celebrex 200 mg daily for 4 months.

At 2 weeks postoperatively, ROM was 3°–109°. At 4 months, the ROM decreased to 6°–90°, and another MUA with an intra-articular corticosteroid injection was performed. Following this, the patient's final ROM was 5°–143°. At 2.8 years follow-up, the patient reported their knee to be much better than before surgery, and they were satisfied (Table 2).

### 2.2. Case 2

A 19-year-old female presented with right knee arthrofibrosis following an ACLR performed 17 months prior at an outside institution. No meniscus repair was performed at the time of surgery. The patient's postoperative ROM was 15°–90°, and she underwent an arthroscopic LOA and MUA 3 months after her initial ACLR. Postoperatively, she utilized PT along with a dynamic stretching splint and CPM. The patient had a persistent limited ROM of 15°–95° at 2 months postoperatively and underwent a repeat LOA and MUA. Postoperatively, the patient reported persistent pain and difficulty ambulating with a decreased ROM of 15°–105°. An MRI revealed an intact ACLR graft, formation of a cyclops lesion, and infrapatellar fat pad scarring with patella baja.

The patient was offered a revision arthroscopic LOA, posterior capsular release (due to extension deficit), MUA, and subsequent one-time low-dose irradiation. EUA revealed an ROM of 15°–110°. Arthroscopic findings, consistent with MRI, confirmed substantial infrapatellar and suprapatellar adhesions and the presence of a cyclops lesion, which were all debrided. Postoperative ROM was 5°–130°. She subsequently underwent same-day irradiation with 700 cgy. The postoperative course included the use of a turnbuckle extension brace, PT, Celebrex 200 mg daily (4 months), and doxycycline 100 mg (4 weeks).

At 2 weeks postoperatively, the ROM was 0°–122°, and at 5 months, the patient maintained an ROM of 0°–110°. At 1.4 years follow-up, the patient reported improvement in pain, satisfaction with the outcome, and successful return to activities.

### 2.3. Case 3

A 36-year-old male presented with right knee arthrofibrosis 1 year after an ACLR with medial and lateral meniscus repair at an outside institution. He underwent a LOA at 3 months postoperatively. The patient had full extension and 120° of flexion after this procedure but began to experience a decreased ROM from 0° to 90° at 10 months postoperatively despite intensive PT. An MRI demonstrated an intact ACLR graft, patella baja, and an effusion.

The patient was offered a revision arthroscopic LOA, MUA, and subsequent one-time low-dose irradiation. EUA revealed an ROM of 4°–85°. Arthroscopy demonstrated suprapatellar adhesions, and MUA after LOA produced an ROM of 0°–100°. The patient then received same-day irradiation to the knee of 800 cgy. The postoperative course included a CPM device, PT, Celebrex 200 mg (4 months), and doxycycline 100 mg (4 weeks).

At 2 weeks postoperatively, ROM was 5°–90°, and this persisted at 3 months, leading to an aspiration and corticosteroid injection. At 8 months, ROM was 5°–100°. At 9.5 months, the patient underwent another LOA and MUA due to diminishing ROM gains. Two months after this procedure, the patient received an aspiration and hyaluronic acid injection. At 1-year follow-up, ROM was full extension to 118° flexion, and the patient reported the knee was slightly better than before surgery, but they were not satisfied and were unable to return to activities such as jogging.

## 3. Discussion

Arthrofibrosis is a debilitating complication in patients undergoing ligament reconstruction, significantly impairing ADLs. The risk of arthrofibrosis is greater in women and patients with prior surgical history or chronic injury [3, 4]. There is limited data regarding adjunctive treatments when adequate improvement is not achieved following standard management. We reported three patients who underwent LOA with low-dose irradiation therapy for recalcitrant arthrofibrosis following ACLR. All three patients reported improved ROM and outcomes compared to prior interventions, would repeat the procedure, and experienced no complications aside from needing additional LOA/MUA.

Managing arthrofibrosis after ACLR remains a challenge, particularly in patients who have undergone multiple treatments without significant improvement. All three patients had ACLR surgery at least 1 year prior and underwent standard treatments with multiple LOA/MUA procedures before presenting at our institution. Notably, these prior failed interventions included aspects of the multimodal management they received at our institution (CPM, Celebrex, doxycycline, and intra-articular corticosteroids). Pertinently, the initial procedures differed among the patients. The 36-year-old male underwent ACLR with bilateral meniscus repairs, showing limited improvement following LOA with irradiation and underwent another LOA and MUA within 1 year postoperatively. Meniscal repair is a reported risk factor for arthrofibrosis, potentially due to a more restrictive rehabilitation protocol compared to an isolated ACLR [16, 17]. Limited preoperative ROM and ACLR within 3 weeks following injury are other reported risk factors for arthrofibrosis [18–20].

At our institution, patients with recalcitrant knee arthrofibrosis following ligament reconstruction undergo a standardized arthroscopic LOA. The technique, previously detailed by Hayden et al., follows a systematic stepwise approach [21]. A superolateral or superomedial portal is first established to debride adhesions in the suprapatellar pouch and intercondylar notch, followed by access to the medial and lateral gutters. If present, the prepatellar fat pad and infrapatellar tether are released from the anterior tibia. A modified Gillquist maneuver is then used to establish a posteromedial portal for capsular debridement, while the posterolateral compartment is addressed through a portal just posterior to the lateral femoral condyle, with care taken to protect the common peroneal nerve [21, 22]. The gastrocnemius belly is used as a landmark to guide posterior capsular resection. Finally, a lateral-to-medial transseptal portal is created to remove adhesions between the PCL and the neurovascular bundle [21, 23, 24].

Postoperative management is designed to maximize early motion and reduce scar formation. Our standardized regimen includes immediate use of turnbuckle orthoses, early PT, CPM during rehabilitation, Celebrex, and doxycycline. Nonsteroidal anti-inflammatory medications like Celebrex have demonstrated efficacy in improving postoperative ROM [25, 26]. Doxycycline is also incorporated due to its inhibition of matrix metalloproteinases and potential antifibrotic effects, though clinical evidence remains limited [27, 28]. Earlier application of orthoses has been associated with better outcomes in patients with restricted knee motion, and although CPM remains controversial in TKA cohorts, studies in ACLR populations suggest it may reduce the incidence of postoperative arthrofibrosis [29–34].

**Figure 2:**
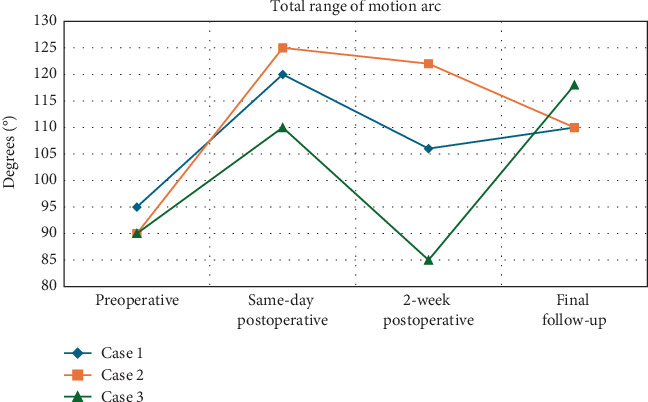
Total range of motion (ROM) arc for patients undergoing revision lysis of adhesions with irradiation treatment.

**Figure 3:**
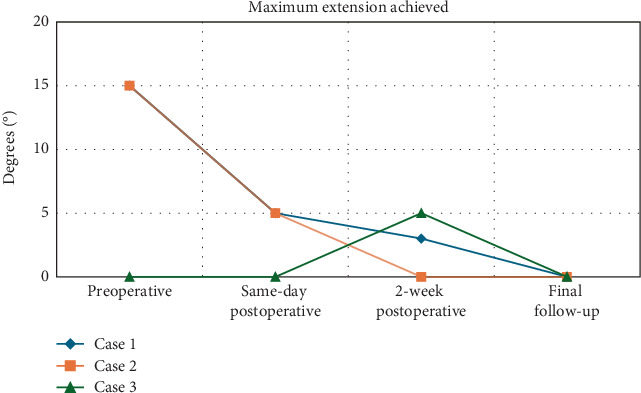
Maximum extension achieved for patients undergoing revision lysis of adhesions with irradiation treatment.

**Figure 4:**
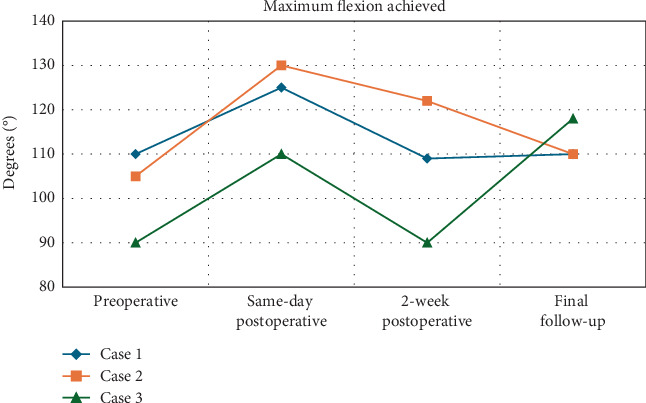
Maximum flexion achieved for patients undergoing revision lysis of adhesions with irradiation treatment.

Adjunctive treatments, such as irradiation, may offer promising outcomes for patients who have not responded to conventional therapies. Our case series of three patients demonstrated total arc ROM improvements of 30°, 20°, and 28°, respectively, along with gains in ADLs and pain relief (Figures 2, 3, and 4). Two of the patients had extension deficits > 10°, which is known to impact ADLs, but both achieved a functional ROM after revision arthroscopic LOA and irradiation [35]. To our knowledge, no studies report outcomes of irradiation therapy for arthrofibrosis following ligament reconstruction. Only a few studies have assessed irradiation for arthrofibrosis in TKA patients. Smith et al. compared postoperative irradiation to no irradiation following revision TKA for arthrofibrosis and found no difference in ROM outcomes, although both groups had significant improvements [13].

While patients in our series did not experience adverse effects, irradiation therapy is not without potential risks. Studies have shown that articular cartilage is at risk of cell apoptosis when irradiated [36]. However, low doses such as between 500 and 3000 cgy have been shown not to induce apoptosis [37–40]. Our cases were exposed to 700–800 cgy and at minimal risk of articular cartilage damage. An additional concern is second malignancy after radiation. The risk of this is expected to be incredibly low. Despite many patients receiving perioperative radiotherapy with resection of heterotopic ossification, only three cases of infield cancers have been reported [41–43]. While few cases have been reported to date, the number of published cases is limited, and long-term data are sparse. Therefore, the risk of radiation-induced malignancy in this population cannot be definitively excluded.

The findings in our series should be interpreted in context, as the multimodal nature of our protocol makes it difficult to isolate the contribution of irradiation. LOA has been shown to independently improve ROM, and two of three patients required additional MUA procedures, highlighting the incremental and multifactorial nature of recovery. All three patients were treated with our standardized perioperative protocol—including structured rehabilitation and pharmacologic adjuncts—which likely contributed to outcomes. Interpretation is further complicated by the fact that these were highly refractory cases, a population that likely responds differently than patients with less severe arthrofibrosis. Overall, the observed gains most likely reflect the combined effect of meticulous LOA, repeated MUA when indicated, perioperative irradiation, and comprehensive rehabilitation. Larger controlled studies are needed to clarify the independent role of irradiation.

## 4. Conclusion

In patients with recalcitrant arthrofibrosis after ACLR who have not responded to standard management, a revision LOA and low-dose irradiation can be safely performed, offering a moderate success rate for the most challenging cases.

## Figures and Tables

**Table 1:** Patient demographics and clinical characteristics.

	**Case 1**	**Case 2**	**Case 3**
Age (years)	35	19	36
BMI (kg/m^2^)	26.6	23.1	25.1
Sex	Female	Female	Male
Laterality	Right knee	Right knee	Right knee
Initial surgery	ACL reconstruction, IT band tenodesis, and MPFL reconstruction	ACL reconstruction	ACL reconstruction with medial and lateral meniscus repairs
Days from initial surgery to revision LOA + irradiation	781 days	237 days	168 days
Number of LOA procedures prior to revision LOA + irradiation	3	2	1
Revision procedure performed with irradiation	Lysis of adhesions, posterior capsular release	Lysis of adhesions and posterior capsular release	Lysis of adhesions
Radiation dose delivered	800 cgy	700 cgy	800 cgy
Preoperative^a^ ROM	15°–110°	15°–105°	0°–90°
Same-day postoperative ROM	5°–125°	5°–130°	0°–110°
Postoperative ROM at 2 weeks	3°–109°	0°–122°	5°–90°
Postoperative ROM at final follow-up	0°–110°	0°–110°	0°–118°
Additional postoperative interventions	Turnbuckle extension brace, continuous passive motion (CPM), physical therapy, Celebrex (4 months)	Turnbuckle extension brace, physical therapy, Celebrex (4 months), and doxycycline (4 weeks)	Continuous passive motion (CPM), physical therapy, Celebrex (4 months), and doxycycline (4 weeks)

^a^Preoperative ROM refers to ROM prior to revision LOA + irradiation.

**Table 2:** Outcomes following low-dose irradiation therapy for recurrent arthrofibrosis post-ACL reconstruction.

	**Case 1**	**Case 2**	**Case 3**
Procedures after revision LOA + irradiation	4 months postoperatively (PO): Manipulation under anesthesia and corticosteroid injection	None	3 months PO: Aspiration and corticosteroid injection 10 months PO: Lysis of adhesions and manipulation under anesthesia 12 months PO: Aspiration and hyaluronic acid injection
Are you satisfied with the results of your knee surgery and radiation?	Yes	Yes	No
Compared to before surgery, would you say that your knee is currently:	Much better than before surgery	Much better than before surgery	Slightly better than before surgery
Did the surgery combined with radiation yield better results than previous interventions (prior surgery, PT, etc.)?	Yes	Yes	Yes
If you could go back and make the decision again, would you still choose to undergo radiation therapy?	Yes	Yes	Yes
VAS pain at rest	1/10	0/10	1/10
VAS pain with use	6/10	2/10	1/10
Tegner Activity Score	Work, heavy labor; competitive sports, cycling and cross-country skiing; recreational sports, jogging on uneven ground at least twice weekly	Work, heavy labor; competitive sports, cycling and cross-country skiing; recreational sports, jogging on uneven ground at least twice weekly	Work, moderately heavy labor; recreational sports, cycling, cross-country skiing, and jogging on even ground at least twice weekly
Lysholm Knee Score	75.0	96.9	75.0
IKDC	51.7	67.8	40.2

## Data Availability

The data that support the findings of this study are available from the corresponding author upon reasonable request.
